# Hydrophobic flavonoids from *Scutellaria baicalensis* induce colorectal cancer cell apoptosis through a mitochondrial-mediated pathway

**DOI:** 10.3892/ijo.2013.1777

**Published:** 2013-01-16

**Authors:** CHONG-ZHI WANG, TYLER D. CALWAY, XIAO-DONG WEN, JACOB SMITH, CHUNHAO YU, YUNWEI WANG, SANGEETA R. MEHENDALE, CHUN-SU YUAN

**Affiliations:** 1 Tang Center for Herbal Medicine Research, and Department of Anesthesia and Critical Care, University of Chicago, Chicago, IL 60637, USA; 2 Department of Medicine, University of Chicago, Chicago, IL 60637, USA; 3 Committee on Clinical Pharmacology and Pharmacogenomics, University of Chicago, Chicago, IL 60637, USA

**Keywords:** *Scutellaria baicalensis*, hydrophobic flavonoids, baicalin, baicalein, wogonin, colorectal cancer, mitochondrial apoptotic pathway

## Abstract

*Scutellaria baicalensis* extract (SbE) has been shown to exert chemopreventive effects on several types of cancer. Baicalin, a hydrophilic flavonoid found in SbE, may have opposing effects that decrease the antitumor potential of SbE against colorectal cancer. In this study, after removing baicalin, we prepared an aglycone-rich fraction (ARF) of SbE and evaluated its anti-proliferative activity and mechanisms of action. The flavonoids found in ARF, baicalin fraction (BF) and SbE were determined by high-performance liquid chromatography (HPLC). The effects of ARF, BF, SbE and representative flavonoids on the proliferation of HCT-116 and HT-29 human colorectal cancer cells were determined by an MTS assay. The cell cycle, the expression of cyclins A and B1 and cell apoptosis were assayed using flow cytometry. Apoptosis-related gene expression was visualized by quantitative real-time polymerase chain reaction (PCR), and mitochondrial membrane potential was estimated following staining with JC-1. HPLC analysis showed that ARF contained two hydrophobic flavonoids, baicalein and wogonin, and that BF contained only baicalin. SbE had little anti-proliferative effect on the colorectal cancer cells; cancer cell growth was even observed at certain concentrations. ARF exerted potent anti-proliferative effects on the cancer cells. By contrast, BF increased cancer cell growth. ARF arrested cells in the S and G2/M phases, increased the expression of cyclins A and B1, and significantly induced cell apoptosis. Multiple genes in the mitochondrial pathway are involved in ARF-induced apoptosis, and subsequent cellular functional analysis validated the involvement of this pathway. These results suggest that removing baicalin from SbE produces an ARF that significantly inhibits the growth of colorectal cancer cells, and that the mitochondrial apoptotic pathway plays a role in hydrophobic flavonoid-induced apoptosis.

## Introduction

Colorectal cancer is the fourth most common type of cancer in the United States and the second leading cause of cancer-related mortality in the Western world (1). Although early diagnosis and rigorous screenings have reduced its incidence in recent years, the prognosis associated with metastatic disease remains bleak. Current treatment for colorectal cancer is surgical resection combined with radiation and chemotherapy. A number of chemotherapeutic agents have been developed from botanical sources (2,3).

Currently, the identification of non-toxic anticancer constituents from botanicals is essential for advancing the treatment of colorectal cancer. Since the composition of herbal extracts is complex, different constituents may have opposing activities (4), which may reduce the beneficial effects of an herb (5). Therefore, the isolation of an active fraction from an herbal extract is essential for the effective use of herbal medicines.

**Figure 1:**
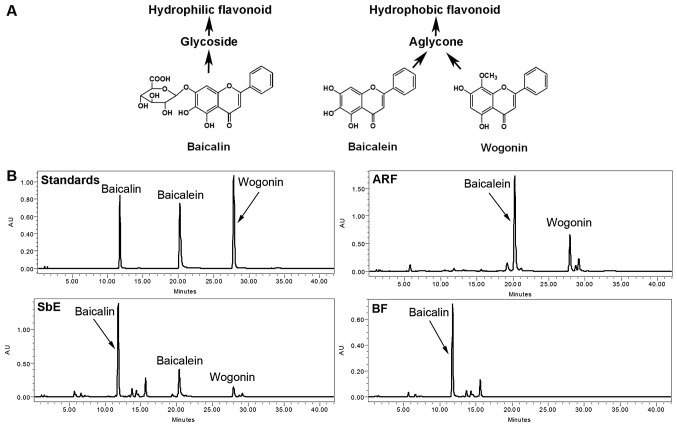
HPLC analysis of flavonoids in *Scutellaria baicalensis* extract (SbE), aglycone-rich fraction (ARF) and baicalin fraction (BF). (A) Chemical structures of the three flavonoids detected. (B) HPLC chromatograms of flavonoid standards and samples.

The root of *Scutellaria baicalensis* (*S. baicalensis*) Georgi (Labiatae) is a widely used herb in the traditional medical systems of China and Japan. The representative constituents of *S. baicalensis* are a group of flavonoids that include baicalin, baicalein, and wogonin (Fig. 1A) (6). *S. baicalensis* extract (SbE) has been used with positive results for inflammatory diseases, allergies, hyperlipidemia and arteriosclerosis (7,8). SbE has also been shown to exert chemopreventive effects on a variety of cancers (9–11). Chemoprevention involves the use of medicines, vitamins, or herbs to delay or prevent the development of cancer.

The effect of *S. baicalensis* on human colorectal cancer remains uncertain; a limited anti-proliferative effect of SbE on human colorectal cancer cells has been reported. Compared to its effect on liver and prostate cancer lines (9,10), the anti-proliferative activity of SbE on human colorectal cancer cells is limited (11,12). Although baicalein inhibits the growth of colon cancer cells (13,14), no such results have been obtained with baicalin, the major constituent of SbE. Therefore, we hypothesized that since baicalin does not significantly inhibit the growth of colorectal cancer cells, the anti-proliferative effect of SbE may be reduced by baicalin, the major hydrophilic flavonoid.

In this study, we prepared an aglycone-rich fraction (ARF), which contains hydrophilic flavonoids, and a baicalin fraction (BF) of SbE. The representative flavonoids in ARF, BF and SbE were determined by high-performance liquid chromatography (HPLC). We then examined the effects of ARF on the growth of human colorectal cancer cells, the cell cycle, apoptosis and apoptotic gene expression. The results of gene expression profiling were validated by a cellular functional assay.

## Materials and methods

### Chemicals

The flavonoid standards, baicalin and baicalein, were obtained from Sigma (St. Louis, MO) and wogonin was obtained from the National Institute for the Control of Pharmaceutical and Biological Products (Beijing, China). All standards were of biochemical-reagent grade and at least 95% pure as confirmed by HPLC. HPLC grade methanol, ethanol, n-butanol and acetonitrile were obtained from Fisher Scientific (Pittsburgh, PA). Milli-Q water was supplied by a water purification system (US Filter, Palm Desert, CA). Trypsin, Dulbecco’s modified Eagle’s medium (DMEM), fetal bovine serum (FBS), and penicillin/streptomycin solution (200X) were obtained from Mediatech, Inc. (Herndon, VA). A CellTiter 96 Aqueous One Solution Cell Proliferation Assay kit was obtained from Promega (Madison, WI). An Annexin V-FITC Apoptosis Detection kit, 7-amino-actinomycin D (7-AAD), FITC-conjugated cyclin A and PE-conjugated cyclin B1 were obtained from BD Biosciences (San Diego, CA).

### Phytochemical isolation and HPLC analysis

The roots of *S. baicalensis* were obtained from Chengde (Hebei, China). The voucher samples were deposited at the Tang Center for Herbal Medicine Research at the University of Chicago. Dried *S. baicalensis* root was ground to a powder and passed through a 40 mesh screen, then extracted with 70% ethanol to obtain SbE. SbE was placed in water and then extracted with ethyl acetate to obtain the ARF. After washing with n-butanol, the water layer was dried to produce the BF. Flavonoid analysis was performed on a Waters HPLC system consisting of a 2960 separations module, a 996 Photodiode Array Detector (Waters, Milford, MA), and Waters Millennium 32 software for peak identification and integration. The separation was carried out on a Phenomenex Prodigy ODS(2) column (150×3.2 mm, 5 *μ*m). Acetonitrile (solvent A) and 0.03% (v/v) phosphoric acid in water (solvent B) were used. Gradient elution started with 15% solvent A and 85% solvent B; it was changed to 28% solvent A for 12 min, to 35% solvent A for 9 min, to 50% solvent A for 7 min, to 95% solvent A for 2 min and then held for 2 min. Finally it was changed to 15% solvent A for 3 min and held for 5 min. The flow rate was 0.8 ml/min and the detection wavelength was set to 280 nm.

### Cell line and cell culture

The human colorectal cancer cell lines, HCT-116 and HT-29, were obtained from the American Type Culture Collection (Manassas, VA) and grown in McCoy’s 5A medium supplemented with 10% FBS and 50 IU penicillin/streptomycin in a humidified atmosphere of 5% CO_2_ at 37°C.

### Cell proliferation analysis

Cells were seeded in 96-well plates (1×10^4^ cells/well). On the second day, various concentrations of ARF, BF, SbE or flavonoids were added to the wells. Cell proliferation was evaluated using an MTS assay according to the manufacturer’s instructions. Briefly, after the cells were treated with drugs for 48 h, the medium was replaced with 100 *μ*l of fresh medium and 20 *μ*l of MTS reagent (CellTiter 96^®^ Aqueous Solution) in each well, and the plate was returned to the incubator for 1–2 h. A 60-*μ*l aliquot of medium from each well was transferred to an ELISA 96-well plate and its absorbance at 490 nm was recorded. Results were expressed as a percentage of the control (solvent vehicles set at 100%).

### Cell cycle, cyclins A and B1 analysis

Cells were seeded in 24-well tissue culture plates (2×10^5^ cells/well). On the second day, the medium was changed, and the cells were treated with ARF, BF or SbE. Cells were incubated for 48 h before harvesting. The cells were fixed gently by adding 80% ethanol before they were placed in a freezer for 2 h. The cells were then treated with 0.25% Triton X-100 for 5 min in an ice bath. The cells were resuspended in 130 *μ*l of PBS. Then, 5 *μ*l of 7-AAD staining solution, 10 *μ*l of cyclin A-FITC and 10 *μ*l of cyclin B1-PE were added to the cell suspension. Cells were incubated in a dark room for 10 min at room temperature and analyzed using a FACScan flow cytometer (Becton-Dickinson, Mountain View, CA) and FlowJo 7.1.0 software (Tree Star, Ashland, OR). For each measurement, at least 10,000 cells were counted.

### Apoptotic analysis

Cells were seeded in 24-well tissue culture plates (2×10^5^ cells/well). On the second day, the medium was changed and ARF, BF or SbE was added. After treatment for 48 h, cells floating in the medium were collected. The adherent cells were detached with 0.05% trypsin. The culture medium containing 10% FBS (and floating cells) was then added to inactivate trypsin. After being pipetted gently, the cells were centrifuged for 5 min at 1,500 × g. The supernatant was removed and cells were stained with Annexin V-FITC and propidium iodide (PI) according to the manufacturer’s instructions. Untreated cells were used as the control for double staining. The cells were analyzed immediately after staining using a FACScan flow cytometer. For each measurement, at least 20,000 cells were counted.

### Reverse transcription and quantitative real-time polymerase chain reaction (PCR)

Cells were treated with 20 *μ*g/ml of ARF or BF for 24 or 48 h. Total RNA was isolated using the RNAeasy kit (Qiagen, Hilden, Germany). First strand cDNA was synthesized from 2 *μ*g total RNA using a SuperScript II First-Strand Synthesis System (Invitrogen, Carlsbad, CA). Quantitative real-time PCR was performed in a reaction volume of 25 *μ*l including 1 *μ*l cDNA. PCR conditions were as follows: 95°C for 15 min followed by 40 cycles at 95°C for 30 sec, 55°C for 30 sec, and 72°C for 30 sec. Glyceraldehyde-3-phosphate dehydrogenase (GAPDH) was used as an internal reference gene to normalize the expression of apoptotic genes. Relative quantification of apoptosis-related genes was analyzed by the comparative threshold cycle (Ct) method. For each sample, the Ct value of the apoptotic gene was normalized using the formula: ΔCt = Ct (apoptotic genes) - Ct (GAPDH). To determine relative expression levels, the following formula was used: ΔΔCt = ΔCt (treated) - ΔCt (control). The value was used to plot the expression of apoptotic genes using the formula 2^−ΔΔCt^.

### Mitochondrial membrane potential (Δψm) analysis

Δψm was estimated after staining with JC-1 (Molecular Probes, Eugene, OR). HCT-116 cells were treated for 24 h with ARF or BF at 50 *μ*g/ml. The control cells were grown in medium containing the same amount of ethanol as the treated cells. The adherent cells were then incubated in 0.5 ml of medium containing JC-1 (2.5 *μ*g/ml) for 20 min at 37°C, and images were taken with a Nikon Eclipse E800 microscope (Nikon Corp., Champignysur-Marne, France).

### Statistical analysis

Data are presented as the means ± standard error (SE) (n=3). A one-way ANOVA determined whether the results had statistical significance. In some cases, the Student’s t-test was used for comparing two groups. The level of statistical significance was set at P<0.05. For the effects of components on HCT-116 or HT-29 cell anti-proliferation, cell cycle, cyclins and apoptotic induction, the significance of the treatment groups vs. the control group was assessed by the Student’s t-test.

## Results

### HPLC analysis of flavonoids in ARF, BF and SbE

As shown in Fig. 1B, the representative constituents of SbE are baicalin, baicalein and wogonin. ARF contains two hydrophobic flavonoids, baicalein and wogonin; the major constituent of BF is baicalin. The concentrations of these flavonoids were calculated using their standard curves. In SbE, the concentrations of baicalin, baicalein and wogonin were 156.7, 51.9 and 14.5 mg/g, respectively. In BF, the concentration of baicalin was 166.8 mg/g, and baicalein and wogonin were not detected. The concentrations of baicalein and wogonin in ARF were 405.4 and 123.5 mg/g, respectively; baicalin was detected only in trace amounts (5.1 mg/g). Although baicalin accounted for 70.2% of the total flavonoids detected in SbE, in ARF, the proportion was <1% (0.96%), suggesting that the quality of the ARF was acceptable.

### Effects of ARF, BF, SbE and flavonoids on the proliferation of colorectal cancer cells

**Figure 2:**
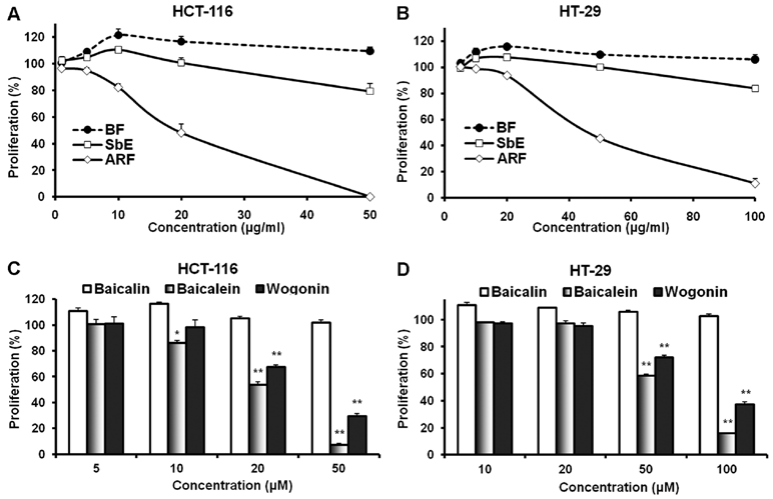
Effects of ARF, related fractions and representative flavonoids on the proliferation of (A and C) HCT-116 and (B and D) HT-26 human colorectal cancer cells. Cells were treated with (A and B) ARF, BF, SbE, or (C and D) flavonoids for 48 h and then assayed using the MTS method. Data are presented as the means ± SE of triplicate experiments. ^*^P<0.05, ^**^P<0.01, vs. control.

We used the human colorectal cancer cell lines, HCT-116 and HT-29, to evaluate the effects of ARF, BF and SbE on cell proliferation. As shown in Fig. 2, the complete extract, SbE, did not inhibit cell growth at concentrations of 1–20 *μ*g/ml for HCT-116 cells and 5-50 *μ*g/ml for HT-29 cells. Of note, at certain concentrations, SbE actually increased cell growth. We then observed the effects of ARF and BF on the proliferation of cancer cells. BF, which contains only baicalin, increased cell growth. In HCT-116 cells at 10 and 20 *μ*g/ml, BF increased cell growth by 21.4±4.5 and 16.6±3.9%, respectively, compared to the control (both P<0.05). By contrast, ARF showed a potent anti-proliferative effect. At 10 and 20 *μ*g/ml, ARF inhibited HCT-116 cell growth by 17.7±2.3% (P<0.05 vs. control) and 51.7±6.2% (P<0.01), respectively. In addition, treatment with 50 *μ*g/ml of ARF inhibited cell growth by 99.7±0.2% (P<0.01) (Fig. 2A). Similar results were observed in HT-29 cells (Fig. 2B). These observations suggest that the effect of SbE is reduced by the BF and that ARF is the fraction of SbE with anti-proliferative activity.

To evaluate the contributions of individual flavonoids on the observed effects of different fractions, the anti-proliferative activities of three representative flavonoids were examined using HCT-116 and HT-29 cells. Among them, two are aglycones (baicalein and wogonin) and one is a glycoside (baicalin). As shown in Fig. 2C, the treatment of HCT-116 cells with 5–20 *μ*M of baicalin for 48 h did not result in a significant anti-proliferative effect; on the contrary, at 5 and 10 *μ*M, baicalin increased cancer cell growth. The two aglycones, baicalein and wogonin, which are hydrophobic flavonoids, showed significant anti-proliferative effects at concentrations of 20–50 *μ*M. The activity of baicalein was more potent than that of wogonin. Similar results were observed in the HT-29 cells (Fig. 2D). Since HT-29 is a multi-drug resistant cell line, the active concentration of fractions and flavonoids in this cell line is higher than the concentration in HCT-116 cells. These results suggest that the anti-proliferative activities of flavonoid aglycones (such as baicalein and wogonin) are significantly higher than those of flavonoid glycosides (such as baicalin).

### Effects of ARF on the cell cycle and expression of cyclins A and B1

**Figure 3:**
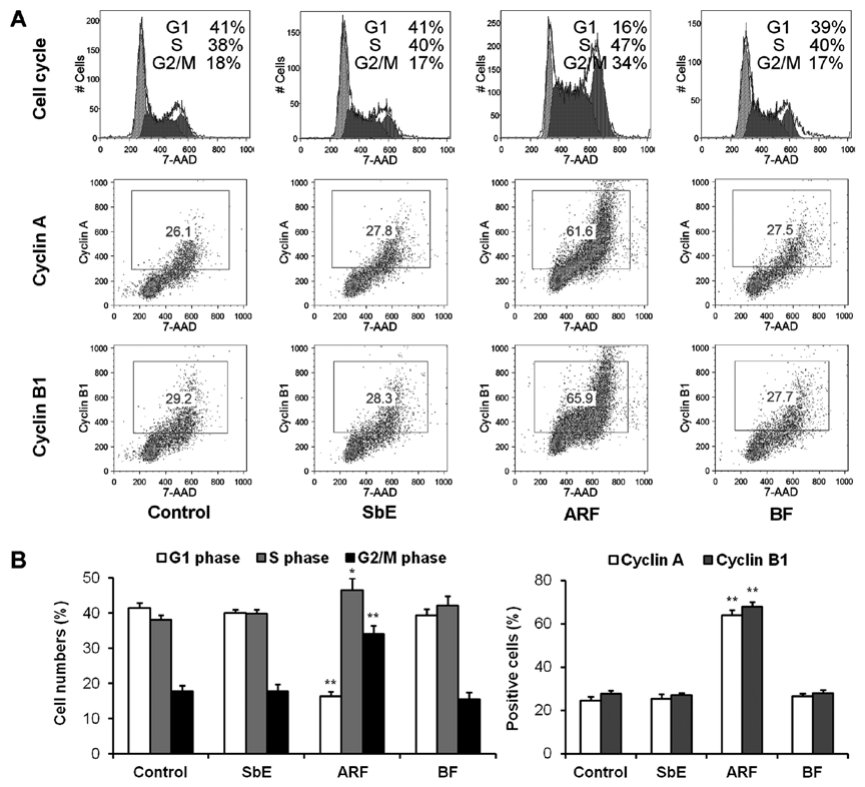
Cell cycle, cyclin A and B1 analysis of HCT-116 cells using flow cytometry. After the HCT-116 cells were treated with 20 *μ*g/ml of ARF, BF or SbE for 48 h, the cells were stained with 7-AAD, cyclin A-FITC and cyclin B1-PE. (A) Typical cell cycle profile and expression of cyclins A and B1. Percentage of cells in the G1, S and G2/M phases, and cyclin A- and B1-positive cells are indicated. (B) Data are presented as the the means ± SE of triplicate experiments. *P<0.05, ^**^P<0.01, vs. control.

After treatment with 20 *μ*g/ml ARF, BF or SbE for 48 h, the cell cycle profile was determined. As shown in Fig. 3, treatment with ARF decreased the number of cells in the G1 phase, and increased the number of cells in DNA synthesis (S) and G2/M phases significantly; SbE and BF showed no effect on the cell cycle.

Cell cycle progression is regulated by the activity of cyclins and cyclin-dependent kinases. Cyclin A is a key regulator of DNA replication and mitosis in the S phase and for passage through the G2/M phase (15). Cyclin B1 plays an important role in the control of the G2-M transition of the cell cycle (16). To elucidate the molecular mechanisms involved in the observed arrest of the cell cycle in the S and G2/M phases, the expression of cyclins A and B1 was determined (Fig. 3). After treatment with 20 *μ*g/ml of ARF, the expression of cyclin A was increased to 64.1±2.3%, compared to 24.5±2.0% in the untreated cells (P<0.01). Compared to the control (27.6±1.8%), the expression of cyclin B1 also increased to 68.0±2.1% (P<0.01). Cyclin A and B1 expression was not increased in the cells treated with SbE or BF. The accumulation of cyclins A and B1 induced by ARF was critical in promoting cell cycle arrest in the S and G2/M phases.

### Effects of ARF on induction of apoptosis

**Figure 4:**
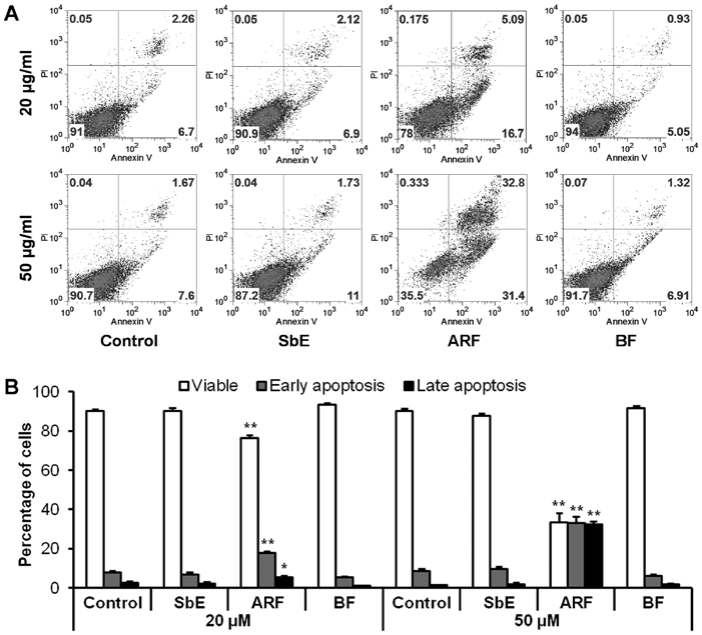
Apoptosis assay using flow cytometry after staining with Annexin V-FITC/propidium iodide (PI). HCT-116 cells were treated with 20 or 50 *μ*g/ml of ARF, BF or SbE for 48 h. (A) Representative scatter plots of PI (y-axis) vs. Annexin V (x-axis). Viable cells are shown in the lower left quadrant, early apoptotic cells are shown in the lower right quadrant, late apoptotic cells are shown in the upper right quadrant, and non-viable cells that underwent necrosis are shown in the upper left quadrant. (B) Percentage of viable, early apoptotic and late apoptotic cells. Data are presented as the means ± SE of triplicate experiments. ^*^P<0.05, and ^**^P<0.01 vs. control.

To determine whether the decrease in cell number observed after treatment with ARF was the result of apoptosis, cells were stained with Annexin V/PI. Annexin V can be detected in both the early and late stages of apoptosis. PI enters the cells in late apoptosis or necrosis. After treatment for 48 h with 20 and 50 *μ*g/ml of ARF, compared to the control (7.8±1.1 and 8.4±1.2%), the percentage of early apoptotic cells was 17.7±0.9 and 33.1±3.3%, respectively (both P<0.01 vs. control) (Fig. 4). ARF clearly induced significant apoptosis in the HCT-116 cells. Treatment with the same concentration of BF or SbE did not induce apoptosis. These results suggest that the anti-proliferative effect of ARF is caused by apoptosis.

### Effects of ARF on apoptotic-related gene expression

We also evaluated the effect of ARF and BF on selected pro-apoptotic genes. Among the selected targets were tumor protein p53 (TP53), tumor protein p53 binding protein 2 (TP53BP2), and four tumor necrosis factor (TNF) family genes: TNF super-family member 2, CD70 molecule (CD70), tumor necrosis factor receptor superfamily member 10a (TNFRSF10A), and tumor necrosis factor receptor superfamily member 10b (TNFRSF10B). As shown in Table I, after treatment with ARF, the expression of TP53 and TP53BP2 was markedly increased, suggesting that ARF induces apoptosis partly through the upregulation of p53. In addition, p53-wild-type cells (HCT-116) were more sensitive to ARF than p53-mutated (HT-29) cells (Fig. 2A and B). Others studies have also demonstrated that TP53BP2 induces apoptosis through the mitochondrial death pathway (17,18).

Another pathway to apoptosis is the death receptor-mediated pathway. The best characterized death receptors and ligands are those of the TNF superfamily (19,20). We determined the effects of ARF on the expression of genes in the TNF ligand (TNF and CD70) and receptor (TNFRSF10A and TNFRSF10B) family. ARF upregulated TNF and CD70 expression, although the expression of TNFRSF10A was not affected. ARF, however, significantly downregulated TNFRSF10B expression (Table I). Based on these results, we cannot confirm the contribution of the death receptor-mediated pathway in ARF-induced apoptosis.

As illustrated in Fig. 2, BF increased colorectal cancer cell growth. We sought to determine whether the expression of selected anti-apoptotic genes is regulated by BF. Since at 20 *μ*M, both the apoptotic induction activity of ARF and cell proli feration exciting activity of BF were observed, we selected this concentration to determine both the pro-apoptotic and anti-apoptotic effects of ARF or BF. As shown in Table I, at 24 h, IGF1R and BCL2 were upregulated by BF; at 48 h, only IGF1R was markedly upregulated. This observation suggests that BF enhances the expression of certain anti-apoptotic genes. By contrast, ARF downregulated IGF1R, BCL2 and BCL2L1 at 24 and 48 h. The Bcl-2 family consists of pro-apoptotic and anti-apoptotic genes, and the balance in expression between these genes helps determine the death or survival of a cell. BCL2 (Bcl-2) and BCL2L1 (Bcl-xL) are two anti-apoptotic genes in the Bcl-2 family (21). Forced downregulation of the anti-apoptotic Bcl-2 family genes results in mitochondrial dysfunction and apoptosis (22). BCL2 and BCL2L1 expression was depressed by ARF, demonstrating that apoptosis is induced at least partly through a mitochondrial mechanism.

### Effects of ARF on Δψm

**Figure 5:**
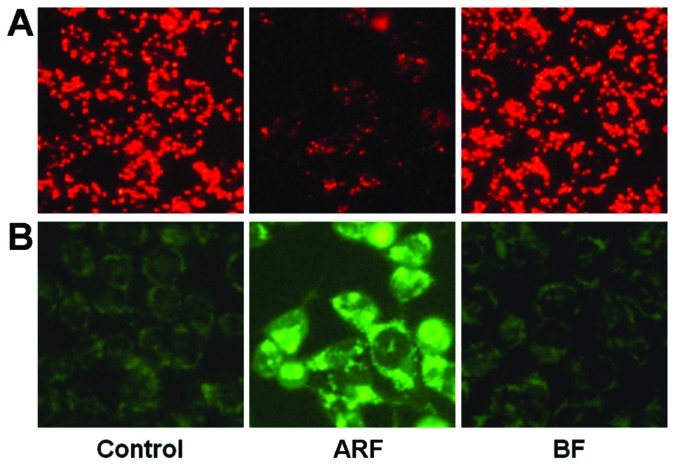
Analysis of the mitochondrial membrane potential (Δψm) after treatment. HCT-116 cells were treated with 50 *μ*g/ml of ARF or BF for 24 h and then stained with JC-1. Red fluorescence (A) represents mitochondria with intact membrane potential. Green fluorescence (B) represents de-energized mitochondria. Images were taken with a fluorescence microscope.

To validate the quantitative real-time PCR data, we performed a cellular functional assay. The spatial variation in Δψm was estimated using the JC-1 probe. This probe accumulates specifically in the mitochondria in varying amounts according to membrane potentials. Organelles with low Δψm accumulate a low number of JC-1 molecules and produce a green fluorescence (485 excitation/535 emission). At high concentrations (high Δψm), the probe aggregates exhibit a red fluorescence (535 excitation/590 emission). The loss of membrane potential is followed by a red to green shift. As illustrated in Fig. 5, untreated HCT-116 cells exhibited red fluorescence. After treatment with 50 *μ*g/ml of ARF, fluorescence shifted from red to green, indicating the loss of mitochondrial function. Compared to the control, treatment with 50 *μ*g/ml of BF did not alter the Δψm. These results suggest that ARF induces apoptosis through a mitochondrial-dependent mechanism.

## Discussion

The clinical management of cancer invariably involves diverse conventional modalities, including surgery, chemotherapy and radiation. The complex characteristics of human cancer may also require alternative management to improve the therapeutic efficacy of conventional treatment and/or the quality of life for cancer patients. Complementary and alternative medicine (CAM) has recently gained attention for cancer management (23–25), since CAM covers a wide spectrum of ancient to modern approaches that expand options for preventing and treating diseases (26,27).

Botanicals have been a valuable source of therapeutic candidates for new compounds since tremendous chemical diversity is found across the millions of species of plants. Since 1961, nine plant-derived compounds have been approved in the US for the treatment of cancer. Several plant-derived anticancer agents, such as flavopiridol, acronycine, bruceantin, and thalicarpin, have been evaluated in clinical trials in the US (28,29). In addition, another 11 anticancer agents are being used in China (28–30). Thus, botanicals have contributed significantly to cancer therapy for the past 30 years, and it is likely that this class of medication will continue to be important in cancer therapeutics.

Over the years, the anticancer activities of SbE and its constituents have been evaluated. Published studies have focused on prostate and liver cancer (9,10,31). The anti-proliferative effect of baicalin was studied in human prostate cancer and human hepatoma cells. The results showed that the threshold for the inhibition of cell growth by 50% (IC_50_) in LNCaP prostate cancer cells was 60.8 *μ*M, and in SK-Hep1 hepatoma cells was 25 *μ*M (32,33). Another major flavonoid in this herb, baicalein, possesses a stronger anti-proliferative effect than baicalin. The IC_50_ of baicalein was 29.8 *μ*M for LNCaP cells and 9.1 *μ*M for SK-Hep1 cells (32,33). The two components differ in chemical structure: in baicalin, the 7-hydroxy group of baicalein is replaced by a glucuronyloxy group. This structural difference between baicalin and baicalein may contribute to the difference in their pharmacological activities (34).

To evaluate the anti-colorectal cancer potential of SbE and its fractions, we determined their anti-proliferative activities. There are several human colorectal cancer cell lines, of which HCT-116 is widely used in laboratory cancer research, and has been a model for cellular pathway studies of chemotherapy on cancer cells (35). HT-29 cells maintain the capacity to conduct phase II metabolism and may conjugate chemotherapeutic agents *in vitro*, whereas HCT-116 cells do not have this ability. Due to phase II metabolism, HT-29 cells exhibit a resistance to many drugs. Thus, we selected these two cell lines (36,37).

The whole extract, SbE, did not exhibit significant anti-proliferative activity. To our surprise, at certain concentrations, SbE actually increased HCT-116 and HT-29 cell growth. Previous reports have demonstrated that the anti-proliferative effect of baicalein is significantly higher than that of baicalin (32,33). In other studies, we observed that sugar moieties on ginsenosides significantly impact their anticancer activity. In general, the anticancer activity is inversely correlated with the number of sugar moieties (38). Therefore, we removed the glycoside, baicalin, from SbE to prepare an ARF and evaluated its potential chemopreventive effect on colorectal cancer.

Of note, ARF exerted a potent anti-proliferative effect on both colorectal cancer cell lines. The BF did not exhibit anti-proliferative activity, and in fact, significantly increased the growth of HCT-116 and HT-29 cells at certain concentrations. We also evaluated the effect of three representative flavonoids on cancer cell growth. Two aglycones, baicalein and wogonin, had significant anti-proliferative activity; the glycoside baicalin did not. At certain concentrations, baicalin even enhanced cancer cell growth. The anti-proliferative effect of SbE was decreased by the presence of baicalin. We may therefore conclude that to enhance anticancer activity, it is necessary to remove baicalin from SbE.

The effect of the herb on cell cycle arrest and the induction of apoptosis in colorectal cancer cells was also evaluated. Potent activity was observed after treatment with ARF, as HCT-116 cells were arrested in the S and G2/M phases, both cyclin A and cyclin B1 were upregulated, and the percentage of apoptotic cells was significantly increased. Since apoptosis is considered an important mechanism in the inhibition of cancer, to further explore the mechanism of ARF-induced apoptosis, we performed expression profiling analysis using quantitative real-time PCR. The results showed that ARF upregulated the expression of TP53, TP53BP2, TNF and CD70 and down-regulated the expression of BCL2, BCL2L1 and TNFRSF10B. Many of these genes are related to the mitochondrial apoptotic pathway. These results were further confirmed by a cellular function assay of the Δψm.

The mitochondrial cell death pathway is mediated by the Bcl-2 family of proteins, a group of anti-apoptotic and proapoptotic proteins that regulate the passage of small molecules through the mitochondrial transition pore. These molecules, such as cytochrome *c*, Smac/Diablo, and apoptosis-inducing factor, activate caspase cascades. ARF treatment led to a loss of Δψm, suggesting that ARF induces apoptosis, at least in part, through the mitochondrial pathway. Of note, BF did not influence the expression of pro-apoptotic genes, instead increasing the expression of anti-apoptotic genes such as BCL2 and BCL2L1.

To explore the possible structure-function correlation, after comparing the effects of baicalin and baicalein on anti-proliferation, we observed whether sugar residues on aglycons decrease or abolish the anti-proliferative activities of the constituent. Similar results were also observed in saponins or ginsenosides of ginseng, another commonly used herbal medicine. The major bioactive constituents in ginseng are ginsenosides and sugar molecules within a ginsenoside have a high impact on tumor cells. Anticancer activities increase with the decrease in the number of sugar moieties. Ginsenosides with four or more sugar molecules (e.g., Rb1 and Rc) show no significant anti-proliferative effects. Rd with three sugar molecules weakly inhibits the growth of cancer cells. Ginsenosides Rg3 (two sugar residues), Rh2 (one sugar residue), IH-901 (one sugar residue), PPT (no sugar residues) and PPD (no sugar residues) showed potent anti-proliferative effects on different types of cancer cells (38). The data from this study suggest that the sugar moiety in baicalin may influence the anti-proliferative activity of baicalein.

In conclusion, to safely and effectively use the botanical components of SbE, we prepared ARF and evaluated its chemo-preventive effects on human colorectal cells. An apoptotic assay and expression profiling of genes in selected pathways revealed that ARF regulated various apoptosis-related genes. Data from our gene expression and cellular functional analyses indicated that the mitochondrial apoptotic pathway is responsible for the anticancer effects of ARF. In addition, since the constituents of SbE are complex, the development of a novel preparation protocol to yield a high content of hydrophobic flavonoids with strong anti-proliferative effects and removal of counteractive or inactive constituents such as baicalin may lead to a significant improvement in the anticancer activity of *S. baicalensis*.

## Figures and Tables

**Table I:** Expression of apoptosis-related genes regulated by aglycone-rich fraction (ARF) and baicalin fraction (BF).

Gene symbol	Quantitative real-time PCR primer sequences	Fold change vs. control			
		ARF 24 h	ARF 48 h	BF 24 h	BF 48 h
Pro-apoptotic genes					
TP53	TGGCATTTGCACCTACCTCAC	0.99	1.25	−0.01	−0.13
	AACTCCCTCTACCTAACCAGC				
TP53BP2	ATTAGAGGACATTTAGCGTGATG	0.60	1.06	0.25	0.17
	AACACTCAACAGGACAGAGAGC				
TNF	AGTTGTGTCTGTAATCGCCCTAC	1.41	2.58	0.47	0.15
	CTAAGCAAACTTTATTTCTCGCCAC				
CD70	GCGTCTCAGCTTCCACCAAG	1.39	3.50	−0.39	0.21
	TGCACTCCAAAGAAGGTCTCATC				
TNFRSF10A	CCACCAGCTAGAGTACATCT	−0.02	0.14	−0.13	−0.01
	TGCTGTCCCATGGAGGTAG				
TNFRSF10B	ATCTGTCTCCCACGTCTGC	−1.12	−2.11	0.10	−1.02
	CCAAGGTCCTCAAGTAGGCAATC				
Anti-apoptotic genes					
IGR1R	ATTCCTGTTATTGCGATATACTCTG	−0.97	−0.33	0.61	1.50
	ACGTTGCCTTAGCTTCAGC				
BCL2	GGCCTTCTTTGAGTTCGGTG	−3.97	−2.07	1.55	0.28
	ACAGGGCGATGTTGTCCAC				
BCL2L1	GCTCCTCATGGTGGGTTCAG	−1.57	−0.59	0.04	0.04
	GCTCCCATAGCTGTTCCTG				

RNA was extracted from the HCT-116 cells following treatment with 20 *μ*g/ml of ARF or BF for 24 or 48 h. Compared to the control, zero represents no change, while positive and negative numbers represent up- and downregulation, respectively.
